# Influenza vaccine uptake among healthcare workers in GCC countries: a narrative review of enablers and barriers

**DOI:** 10.3389/fpubh.2026.1793005

**Published:** 2026-08-11

**Authors:** Syed Abid Ali, Silvija Ille, Jayakumary Muttappallymyalil, Jayadevan Sreedharan, Mehzabin Ahmed

**Affiliations:** 1 First Faculty of Medicine, Charles University, Prague, Czechia; 2 Oxford University Hospital, Oxford, United Kingdom; 3 Department of Community Medicine, College of Medicine, Gulf Medical University, Ajman, United Arab Emirates; 4 Department of Biomedical Sciences, College of Medicine, Gulf Medical University, Ajman, United Arab Emirates

**Keywords:** GCC countries, healthcare workers, influenza vaccine barriers, influenza vaccine enablers, influenza vaccine uptake

## Abstract

**Introduction:**

Influenza is a contagious viral infection and a significant seasonal threat to public health. Vaccinating healthcare workers (HCWs) is a critical strategy for protecting vulnerable patients and the healthcare workforce. In the Gulf Cooperation Council (GCC) countries, vaccine uptake is <75%, despite the healthcare promotion initiatives and free vaccination campaigns. To guide future initiatives, this review aims to evaluate the evidence regarding influenza vaccine uptake among HCWs in the GCC, as well as the perceived enablers and barriers.

**Methods:**

A comprehensive search was conducted across PubMed, Scopus, Google Scholar, and ScienceDirect for relevant studies published between 2010 and 2025. This review includes studies addressing influenza vaccine utilization and its determinants among HCWs in GCC countries.

**Results:**

Vaccine uptake rates across the GCC vary widely, from 36.9% in a study from Bahrain to 88.3% in Saudi Arabia during the Hajj season. Although higher than the global average, vaccine uptake in the GCC countries (about 51%), is still below the WHO target.

**Discussion:**

The desire for self-protection and protection of patients and family members were the commonly cited vaccination enablers. Institutional factors, including mandatory policies, employer recommendations, free on-site access, and targeted awareness campaigns, were strongly associated with higher uptake. Conversely, barriers such as fear of side effects, doubts about vaccine efficacy, misconceptions about the necessity of vaccination, and logistical constraints such as lack of time was commonly cited. Influenza vaccine coverage among HCWs in the GCC is suboptimal and highly variable. Mandatory institutional policies and accessible campaigns have proven to be effective enablers. To achieve WHO targets, future strategies focusing on mitigation of specific barriers such as educational efforts to counter misinformation, increase the convenience of vaccination and improve access for all HCWs across healthcare cadres.

## 1 Introduction

Influenza, commonly known as the flu, is a common contagious viral infection that spreads through the respiratory route. Influenza viruses are single-stranded RNA viruses and are of types A, B, C, and novel type. Type A affects both animals and humans, and is implicated in epidemics and pandemics, being the most virulent type, mutating more frequently compared to the others. Based on the combination of its surface proteins, hemagglutinin (HA) and neuraminidase (NA), Type A influenza virus is classified into 18 HA and 11 NA types (1). Frequent mutations in the genes of Type A viruses are responsible for antigenic variation promoting immune evasion; slow changes over a long time are called antigenic drifts, while sudden changes are called antigenic shifts (2). The virus A (H1N1) and A (H3N2) are currently prevalent (3). Type B is a less virulent virus with slower mutation rate and commonly affects children and people in crowded facilities; the common subtypes are the B/Victoria and B/Yamagata lineages (1, 3, 4). Type C is the mildest and rarely poses a public health problem. The novel type is mostly found in animals but can infect humans (3). While Types A and B commonly cause seasonal flu epidemics, only Type A is implicated in causing pandemics (3). The Global Influenza Programme, conducted by the WHO, monitors, identifies, and recommends measures to prevent epidemics, by advocating suitable vaccine compositions and timings of the vaccination campaigns, according to the geographic location of the countries (3). Primary prevention of flu is by administration of a vaccine prepared using a combination of strains predicted to cause the illness. Vaccine preparation requires an elaborate process, undertaken by the WHO Collaborating Centres for Influenza, involving the identification of about 2000 current strains and monitoring antigenic changes (5, 6). The current WHO recommendation is the trivalent vaccine, which includes H3N2, H1N1, and the Victoria lineage; the Yamagata lineage has been removed as its prevalence has drastically decreased since the COVID-19 pandemic (7).

The Gulf region in the Middle East is unique in terms of its climatic conditions, with high heat and humidity for most of the year. This contributes to year-round possibility of virus transmission; other contributing factors include the density of the urban population and the region being a hub of global travel during the mass gatherings of pilgrimages (8, 9). Mismatches between circulating influenza strains and available vaccine formulations may occur in the Northern Hemisphere, thus compromising vaccine effectiveness even before regional epidemics peak (8). Healthcare workers (HCWs), defined by the WHO as “all people engaged in activities whose primary intent is to enhance health,” working in such situations are also at increased risk [(10), p2].

Influenza infections among HCWs impact not only the individuals but also the healthcare delivery and patient safety (11, 12). Though limited data is available on the full extent of the effect on HCWs, reports on the severity patterns of the illness offer some insight. Studies from Saudi Arabia, Oman, Bahrain, and the United Arab Emirates highlight the disproportionate burden on vulnerable populations, such as adults ≥65 years, patients with co-morbidities, children, and pregnant women, who experience a high influenza-associated mortality rate (13–17). Given the high prevalence of comorbidities such as diabetes (affecting 8–22% of the adult GCC population) and cardiovascular diseases among both patients and HCWs, infections in clinical settings carry elevated risks for severe complications (13). The target for influenza vaccination among HCWs is set at ≥75% by the WHO, however, in Middle Eastern countries it is low, reaching about 51%, a finding that is significantly better than Asia (28.5%) and Africa (6.5%) and the global rates of 41.7%, but lower than the Americas (67%) (14–16). A unique characteristic of the HCWs working in the GCC is their migrant and multinational composition, which may have a bearing on vaccination rates due to the transient nature of employment, their country of origin, and the level of awareness and practices followed regarding vaccination (14). Another important determinant is the availability of the vaccine at the work site and encouragement by governing bodies, which fosters a pro-vaccination stance (15). Advocacy of vaccination guidelines for the and promotion of the practice of vaccination, by way of making it available for free at the start of the season, and at the worksite are steps that help improve vaccination rates (16, 17). A unique feature of the influenza virus is its ability to constantly mutate, which makes it difficult to predict the efficacy of the vaccine; the vaccines are prepared as a combination of likely strains to be prevalent in a given season, ascertained by constant monitoring of the prevalence of the specific subtypes before prediction, a process that takes at least 6–8 months (6). Seasonal vaccine efficacy can get as low as about 20% due to mutation in viral antigens and needs to be repeated each season for the very same reason (5, 6).

While several studies have been conducted on vaccination among the general population and its advocacy by the HCWs, fewer studies have been conducted among HCWs in the Gulf Cooperation Council (GCC) countries. The utilization of the influenza vaccine among HCWs in GCC countries remains variable and below the World Health Organization (WHO) target of 75% coverage, despite significant regional investment in vaccination campaigns and free vaccine availability (18, 19). Given that morbidity and mortality due to seasonal influenza is high among patients with co-morbidities, and HCWs who tend to such patients may be the source of influenza transmission, it is of critical importance that the HCWs are vaccinated (11–13). With a WHO goal of at least 75% vaccination among priority groups, HCWs included, it is important that the factors that promote/ motivate or pose barriers must be identified and targeted to achieve the goal (20). Studies from Saudi Arabia, the United Arab Emirates, Qatar, Oman, and Kuwait provide comprehensive insight into uptake rates, determinants, and barriers. However, a review evaluating and collating the findings of such studies is lacking for the region. The findings from this review will help guide efforts, by clinicians and policy makers, to invest, plan and implement programs to remove the barriers causing vaccine hesitancy and improve the vaccination rates among the HCWs (11).

## 2 Materials and methods

**Figure 1:**
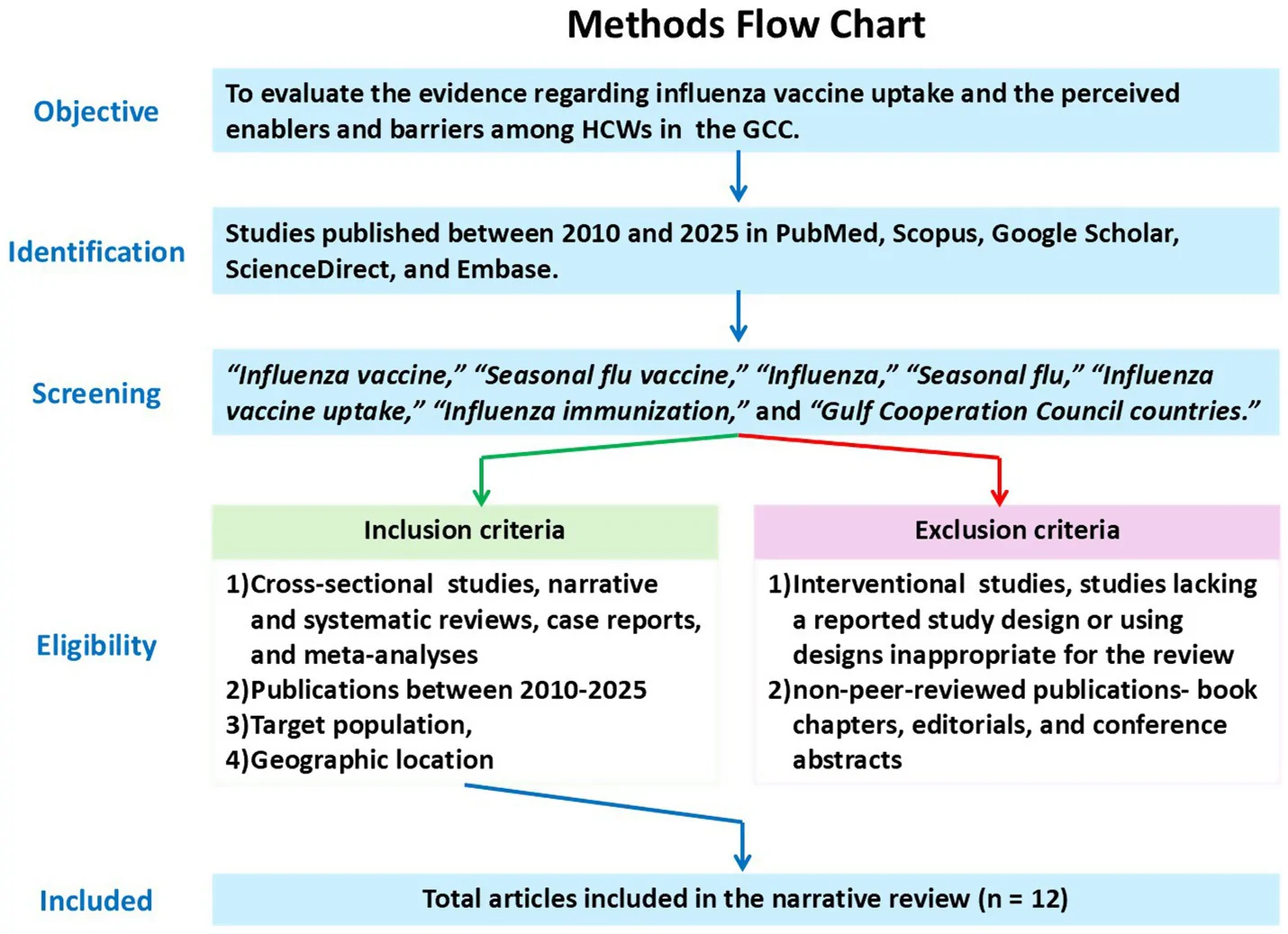
Methods flow chart for selection of studies on influenza vaccine uptake among HCWs in the GCC.

This review was conducted as a narrative review. To enhance transparency and methodological clarity, elements of a structured approach were adopted for literature search, study selection, and data extraction. However, this study does not represent a full systematic review, as it did not aim to include all available literature and no formal quality assessment of included studies was performed. Given the heterogeneity of the included studies, the findings were synthesized narratively (see Figure 1).

### 2.1 Search strategy

The authors conducted a structured literature search of electronic databases including PubMed, Scopus, Google Scholar, ScienceDirect, and Embase for studies published between 2010 and 2025. This search encompassed all years of publication up to the specified date to ensure a comprehensive capture of relevant literature. To complement the electronic search, reference lists of all included studies were manually screened to identify additional eligible publications that might not have been retrieved through the database search.

The search strategy was developed using appropriate Medical Subject Headings (MeSH) and free-text terms, such as *“Influenza vaccine,” “Seasonal flu vaccine,” “Influenza,” “Seasonal flu,” “Influenza vaccine uptake,” “Influenza immunization,”* and *“Gulf Cooperation Council countries.”* Boolean operators and database-specific filters were applied where applicable to refine and maximize the sensitivity of the search.

Studies were included if they specifically addressed influenza vaccine utilization and its determinants among HCWs in GCC countries. Following retrieval, all identified articles underwent a structured review process in two stages: title and abstract screening to exclude irrelevant studies, followed by full-text review to confirm eligibility. This ensured that only the most relevant and those fulfilling the inclusion criteria were incorporated into the final review.

### 2.2 Study selection and data extraction

Following the literature search, all retrieved articles were imported into Mendeley software, where duplicate records were identified and removed. As part of this narrative review, a structured review approach was adopted to enhance transparency and consistency. The research team reviewed titles and abstracts to identify potentially relevant studies, which were then subjected to full-text assessment for eligibility. Decisions regarding inclusion were made through discussion and consensus among the authors. While efforts were made to ensure a transparent selection process, the approach was applied within the context of a narrative review rather than a formal systematic review.

In total, 12 studies met the inclusion criteria and were incorporated into the review. The included studies represented a diverse range of designs, including cross-sectional studies, case reports, and meta-analyses. Case reports were considered when they provided contextually relevant information on vaccine uptake or hesitancy. Meta-analysis were considered to obtain quantitative data to contextualize the findings. Where a meta-analysis included studies that were also independently cited in this review, the primary studies were used for data extraction to avoid double-counting evidence. The inclusion criteria were based on the year of publication, study design, target population, and geographic location of the study. Specifically, studies were included if they provided relevant data within the scope of the research question, were published within the specified timeframe (2010–2025), and adhered to acceptable study designs stated. Studies reporting general population data were included solely for contextual comparison and did not contribute to the primary conclusions on healthcare worker vaccine uptake and determinants.

Exclusion criteria were also clearly defined. Interventional studies, those lacking a reported study design or those using designs deemed inappropriate for the review, were excluded. Similarly, non-peer-reviewed materials such as book chapters, editorials, and conference abstracts were excluded to ensure the reliability and scientific rigor of the evidence base. This structured approach ensured that only relevant studies were included, thereby strengthening the validity of the findings.

## 3 Results

The extent of research done on influenza vaccines uptake and its determinants is quite variable among the GCC countries with Saudi Arabia leading the number of published articles.

### 3.1 Vaccine uptake

The details of the studies describing vaccine uptake are presented in Table 1. Vaccine uptake notably showed considerable variation across the GCC. In Saudi Arabia, coverage has improved in certain institutional contexts but seems inconsistent across age, gender, profession, and years of experience (21). A cross-sectional study in Arar city showed vaccination rates of 55.9% among HCWs, with higher compliance among physicians (65.3%) and nurses (55.9%) compared with other HCWs (33.3%) (21). Similarly, in Southwestern Saudi Arabia, the vaccine uptake was found to be 45.5%; younger individuals, unmarried staff, and technicians had a lower uptake (22). Vaccination results in lower influenza prevalence, thereby reducing healthcare costs (23). A mandatory vaccination directive from the Ministry of Health at King Abdullah Medical City in Makkah resulted in a rise in the uptake to 88.3% during the 2014/2015 Hajj season; the uptake in the two preceding years was 61.2 and 54.5% (24, 25). However, in 2019, uptake among HCWs assigned to serve the Hajj pilgrims, in multiple primary health centres in and around Makkah, was 76%; 24% HCWs had not been vaccinated before attending, with pharmacists and other cadres showing the lowest compliance (24). Over time, uptake in Saudi Arabia increased from 34% in 2008–2009 to 38% in 2012–2013, and to more than 59% by 2020; the uptake during the COVID-19 pandemic season (2020) fell slightly to 59% (26). Acceptance studies showed that while self-protection was the strongest motivator, misconceptions regarding the importance of vaccinations and its safety posed as deterrents (27, 28).

**Table 1:** Details and key findings of studies on seasonal influenza vaccine uptake among healthcare workers in GCC countries.

Authors	Yr. of Publ.	Country	Study design	Key findings of influenza vaccine uptake	Search engine
Alenazi et al.,	2018	KSA	Cross-sectional survey (204 HCWs)	Uptake 55.9% (physicians 65.3%, nurses 55.9%, others 33.3%); perceived severity doubled compliance; barriers included discomfort with injections and adverse effects.	PubMed/Electronic Physician
Elawad et al.,	2017	Qatar	Cross-sectional vaccination campaign (*n* = 4,700, 22 centers)	Uptake 77%; highest among aides (98.7%), technicians (95.2%); lowest among pharmacists (73.2%) and physicians (84%); campaign success linked to free availability and intensive communication.	MDPI Vaccines
Barqawi et al.,	2021	UAE	Cross-sectional (*n* = 417 HCPs)	Uptake 54.2%; only 38.9% vaccinated annually; barriers: doubts about effectiveness (42%), side effects (39.3%); motivators: self-protection (60.4%), reducing absenteeism (20.9%).	Pharmacy Practice (Scopus/PubMed)
Alkathlan et al.,	2021	KSA	Web-based cross-sectional survey (*n* = 424)	Uptake rose 2017–2019 (45 → 62%) then dropped to 59% in 2020; predictors: >40 yrs., female, non-Saudi, nurses; intention for 2021 higher (79%).	DovePress/PubMed
Rabaan et al.,	2020	KSA	Questionnaire survey (*n* = 633 HCWs)	Uptake increased from 13.3% (2010–14) to 44.1% in 2015; motivators: self/family protection; barriers: not considering vaccine necessary, safety concerns.	Le Infezioni in Medicina
Alrashdi et al.,	2022	KSA	Cross-sectional survey (*n* = 200 HCWs)	Uptake 41% (2016 season); non-Saudis and clinical staff more likely to vaccinate; positive beliefs about vaccine safety and efficacy strongly associated.	Gavin Publishers
Haridi et al.,	2017	KSA	Cross-sectional survey (*n* = 447 HCWs)	Uptake 88.3% (2014/15 season), improved after MOH mandatory vaccination policy; main motivator self-protection (81.5%); avoidance due to misconception “vaccine causes flu” (38.5%).	Journal of Hospital Infection (Elsevier)
Al Awaidy et al.,	2020	Oman	Cross-sectional (*n* = 390 HCWs)	Uptake 60% (2018/19 season); higher among women and doctors/nurses; motivators: self-protection and protecting community; barrier: side effects (33.6%).	MDPI Vaccines
Awadalla et al.,	2020	KSA	Cross-sectional (*n* = 312 HCWs)	Uptake 45.5% (2017/18 season); risk factors for non-vaccination: <40 yrs., single, technicians, poor knowledge; motivators: self-protection (77.5%), family protection (62.7%).	Human Vaccines and Immunotherapeutics
Keerthy and Roets,	2025	UAE	Cross-sectional survey (*n* = 1,018 HCWs)	Uptake 82%; motivators: self-protection, employer policy, protecting family/patients; barriers: inconvenient timing, fear of injections, doubts on effectiveness.	Southern African Journal of Infectious Diseases
Alqahtani et al.,	2022	KSA	Cross-sectional (*n* = 760 HCWs)	Uptake 76% during Hajj 2019; older staff, physicians, non-Saudis more compliant; barriers: concerns on side effects (46%), misconceptions about efficacy (44%).	Middle East Journal of Family Medicine
Alzayani et al.,	2024	Bahrain	Cross-sectional (*n* = 502 adults at PHCs)	Uptake 36.9% in 2022; males more willing than females (43.2% vs. 31.3%); non-Bahrainis more willing than Bahrainis (51.5% vs. 34.6%); 66.9% would recommend to others; determinants: sufficient information (AOR 2.16), belief that vaccine reduces spread (AOR 7.66).	Emerald Insight (Arab Gulf Journal of Scientific Research)

KSA, Kingdom of Saudi Arabia; UAE, United Arab Emirates; H, Hospital.

In Qatar, a concerted vaccination campaign in 2015 raised uptake among staff of the Primary Health Care Corporation to 77%, exceeding the 60% target (29). Uptake varied by profession, with aides (98.7%) and technicians (95.2%) showing the highest rates, while physicians (84%) and pharmacists (73.2%) had lower uptake. This was a major improvement over the 2014 baseline of only 35% (29).

In the United Arab Emirates, earlier data reported by Abu-Gharbieh et al. (30) demonstrated poor uptake with only 24.7% coverage (20, 30). However, a more recent multi-emirate study found an uptake of 54.2%, with only 38.9% of vaccinated HCWs being consistent in adhering to annual revaccination (31). A study covering hospitals in Abu Dhabi reported an uptake of 82% among staff, still below the CDC target of 90%. Physicians and nurses had the highest uptake with 96.5 and 79% respectively, while pharmacists showed the lowest adherence rate of 47.8% (32).

In Oman, the uptake among HCWs in the South Al Batinah governorate during 2018–2019 was 60%, with higher coverage among women and those in general medicine, emergency, or intensive care departments. Nurses reported 52% uptake, and 40% of HCWs, remained unvaccinated, despite the provision of free vaccines, citing side effects as the main barrier (33). Earlier regional data reported 46.4% uptake in Oman (21). In Kuwait, uptake has been consistently higher than in other GCC countries, with 67.2% of HCWs being vaccinated as noted in a multi-country survey (21). However, more comprehensive national data from Kuwait is limited. Thus, Saudi Arabia, Qatar, and Kuwait have a moderate to high uptake in specific studies or campaigns. However, the general pattern across the GCC indicates suboptimal adherence.

The uptake across the GCC can be considered heterogeneous. The coverage in Saudi Arabia varies widely across settings and seasons, from roughly 41 to 88% depending on year and policy context, with implementation of mandatory programs resulting in the highest levels. Qatar’s coordinated Public Health Care Corporation (PHCC) campaign reached 77% in 2015. Recent studies from the United Arab Emirates report moderate uptake in multi-emirate samples and high uptake in a single Abu Dhabi hospital, though regular annual adherence remains a challenge. Oman’s recent governorate-level survey showed 60%uptake. A study conducted across three GCC countries (Kuwait, Oman, and the United Arab Emirates) as a survey of vaccination rates in the same time period showed Kuwait to have 67.2% while Oman and United Arab Emirates showed 46 and 27%, respectively, (30). Key motivators include self-protection and protection of patients and family. Interventions such as mandatory policies, free on-site access, and targeted communications and awareness campaigns are enablers that are associated with higher uptake, as demonstrated by Saudi Arabia’s mandatory campaigns and Qatar’s public healthcare initiative. On the other hand, barriers to getting vaccinated include concerns over side effects, misconceptions about vaccine necessity, lower perceived need, doubts about effectiveness, and logistical constraints related to time or access.

### 3.2 Vaccination enablers

The details of the studies detailing the potential enablers to vaccine uptake are given in Table 2. Vaccine hesitancy is influenced by determinants that may be based on contextual, collective or individual factors. Socio-cultural determinants, environmental factors, historic precedents, health system, institutional, economic, and political influences provide contextual influence. While access to vaccination, vaccine safety and effectiveness are collective determinants, attributes such as age, level of education, socio-economic status, personal beliefs and practices are personal determinants. Depending on the influences these determinants exert, they act as enablers or barriers to vaccination acceptance (34). Enablers of influenza vaccine uptake among HCWs in the GCC countries are multifactorial and influenced by individual, institutional and social determinants. The enabling factors identified in studies from the GCC countries are considered as individual factors, institutional and policy determinants, and socio-cultural influences.

**Table 2:** Details and key findings of studies on potential enablers of seasonal influenza vaccine uptake among healthcare workers in GCC countries.

Authors	Year of Publ.	Country	Study design	Key findings of enablers	Search engine
Elbarazi et.al	2021	UAE	Qualitative study (33 HCPs, Al Ain)	Trust in system, affordability, availability, mandatory policies, and cultural/religious beliefs enabled uptake despite hesitancy towards influenza vaccine.	Taylor and Francis Online (Human Vaccines and Immunotherapeutics, PubMed)
Keerthy and Roets,	2025	UAE	Cross-sectional survey (*N* = 1,018 HCWs, Abu Dhabi hospital)	Uptake 82%; motivators were self-protection (95.6% physicians), family/patient protection, employer recommendations, and mandatory policies.	Southern African Journal of Infectious Diseases (AOSIS, PubMed)
AlMarzooqi et.al	2018	UAE	Cross-sectional survey (*N* = 431 primary HCPs, Dubai)	Uptake 53.4%; motivators: self-protection (94.8%), patient protection (62.6%), family protection (53.5%); knowledge strongly correlated with uptake.	Taylor and Francis Online (Human Vaccines and Immunotherapeutics, PubMed)
Rehmani and Memon	2010	KSA	Cross-sectional study (*n* = 512 HCWs)	Uptake 34.4%; motivators: self-protection (95%), patient protection (64.2%), recommendation by supervisors (24%) risk awareness (88.6%)	PubMed (Vaccine journal)
Awadalla et.al	2020	KSA	Cross-sectional survey (*N* = 312 PHCWs, Abha)	Uptake 45.5%; motivators: risk awareness (77.5%), family protection (62.7%), infection control instructions (60.6%); knowledge gaps significantly associated with non-uptake.	Taylor and Francis Online (Human Vaccines and Immunotherapeutics, PubMed)
Haridi et.al	2017	KSA	Cross-sectional survey (HCWs, tertiary hospital in Makkah)	Uptake 88.3% in Hajj season with mandatory policy; motivators were self-protection, patient protection, improved productivity; barriers included perceived side effects.	Elsevier (Journal of Hospital Infection, PubMed, ScienceDirect)
Elawad et.al	2017	Qatar	Intervention campaign in 22 primary healthcare centers	Uptake increased to 77% after campaign; education and institutional promotion key enablers.	MDPI (Vaccines, PubMed, Scopus)
Alhammadi et.al	2015	Qatar	Cross-sectional survey (223 pediatric HCPs)	Uptake 67.7%; 70% recommended vaccine to patients; motivators included self and patient protection.	Elsevier (Vaccine, PubMed, ScienceDirect)
Abu-Gharbieh et.al	2010	UAE, Kuwait, Oman	Cross-sectional survey (*N* = 993 HCWs, multi-country)	Uptake rates: UAE 24.7%, Kuwait 67.2%, Oman 46.4%; motivators were self-protection and institutional recommendations.	International Journal of Medical Sciences (PubMed, DOAJ)
Alzayani et.al	2024	Bahrain	Cross-sectional survey (*N* = 502 adults, primary care centers)	Uptake 36.9%; 69.9% willing to recommend; knowledge doubled uptake (AOR = 2.16), belief in community protection increased odds ~8-fold (AOR = 7.65).	Emerald Publishing (Arab Gulf Journal of Scientific Research, PubMed)

KSA, Kingdom of Saudi Arabia; UAE, United Arab Emirates.

#### 3.2.1 Individual factors

The most frequently cited individual enabler is the awareness regarding the importance of vaccination to protect oneself and contacts such as family members or patients. However, the extent varies, with higher vaccination rates in settings with mandatory campaigns. A study conducted in the Emirate of Dubai reported that 94.8% of HCWs cited the main reason for receiving the influenza vaccine was for self-protection, while 62.6% cited their patients, and 53.5% cited their household contacts (35). Similarly, in the Emirate of Abu Dhabi, 95.6% of physicians, 72.4% of nurses, and 87.5% of physiotherapists reported self-protection and protecting patients as their main motivators (32). Similarly, in Saudi Arabia 77.5 and 95% of participants in two separate studies, voted for self-protection and 62.7% for family members (22, 36). In Qatar, HCWs also emphasized protection of patients and family as key enablers, with vaccination rates reaching 77% following targeted campaigns (37, 38). In Dubai, 65.3% of HCWs with good knowledge accepted the vaccine compared to lower uptake among those with lower awareness (35).

#### 3.2.2 Institutional and policy determinants

Institutional and policy-level enablers are also significant across GCC countries. Mandatory vaccination requirements, such as annual influenza immunization for HCWs in many United Arab Emirates facilities, have been identified as strong enablers of vaccination (34). Employer recommendations also play a critical role, with 92.1% of physicians and 54.4% of nurses in Abu Dhabi reporting that they were vaccinated following employer encouragement (32). Campaigns organized by health authorities, such as the Dubai Health Authority and the Qatari Ministry of Public Health, boosted uptake. Qatar showed an increase in vaccination from 28–37% to over 64% and Dubai showed that 45.2% of the vaccinated HCWs (out of 53.4%) stated public health recommendations as their reason (35, 37). Accessibility and affordability are critical enablers, with provision of free vaccine, on-site at healthcare facilities, and campaigns offering mobile vaccine carts in the United Arab Emirates and Qatar, making it less time-consuming and more convenient (34, 35).

In Saudi Arabia, uptake increased to 88.3% during Hajj after implementing mandatory vaccination strategies and financial incentives in Makkah (23, 25). Thus, national and institutional policies to make the vaccine available for free, resulted in vaccination rates rising to 77% with greater compliance from older HCWs (29, 36, 37, 39). Data on influenza vaccine uptake and determinants among HCWs in Bahrain is not available; however, the national immunization program provides free influenza vaccines as its uptake in the general population is otherwise suboptimal at 36.9%; recommendations from HCWs were a powerful enabling factor, and 69.9% of participants were willing to advise others (40). Thus, educational awareness campaigns are strongly correlated with higher uptake plays a strong positive role to improve knowledge and clarify misconceptions.

#### 3.2.3 Sociocultural influences

Social and cultural influences may increase uptake by promoting understanding and trust in government policies, belief in societal responsibility, and religious encouragement to protect health, as cited by Emirati HCWs (34). Peer influence within hospital settings, such as role-modelling by physicians, serves as a positive reinforcer reported in studies conducted in Abu Dhabi and Dubai (34, 35).

In summary, the general enablers of influenza vaccine uptake among HCWs in GCC countries include self-protection and protection of patients and families; institutional and governmental policies mandating or recommending vaccination; free availability and ease of access at workplaces; targeted educational campaigns; reinforcement through social and cultural values such as trust in authorities and religious beliefs; and social influence from colleagues and employers. While country-specific uptake rates vary with highest rates observed during contextual campaigns such as Hajj season and mandatory vaccination drives. The consistent enablers across the GCC countries highlight that effective institutional policies combined with cultural reinforcement and education are central to improving and sustaining influenza vaccine utilization among HCWs.

### 3.3 Vaccination barriers

The studies highlighting the key barriers to vaccine uptake are given in Table 3. The uptake of influenza vaccines is persistently challenged by barriers, despite easy and free availability in most GCC countries. Studies show that the overall flu vaccination rates among HCWs are suboptimal and are highly variable among member countries and depend on the country, the year of study, and the healthcare setting (22, 30, 36, 40).

**Table 3:** Details and key findings of studies on potential barriers to seasonal influenza vaccine uptake among healthcare workers in GCC countries.

Authors	Year of Publ.	Country	Study design	Key findings of barriers	Search engine
Elbarazi et al.	2021	UAE	Qualitative study (33 HCPs, semi-structured interviews)	Hesitancy toward influenza vaccine, perceived low efficacy, fear of side effects, distrust of pharma, misinformation on social media, lack of training to address hesitancy	PubMed/Taylor and Francis
Keerthy and Roets	2025	UAE	Cross-sectional (*n* = 1,018 HCWs, tertiary hospital)	Barriers included inconvenient vaccination times (27.9% nurses), fear of injections (up to 40% in respiratory therapists), perceived ineffectiveness, lack of hospital follow-up, limited policy awareness	PubMed/SAJID
AlMarzooqi et al.	2018	UAE	Cross-sectional survey (*n* = 431 HCPs)	46.6% unvaccinated; main barriers: belief of not being at high risk (39.8%), lack of time (28.9%), concerns about side effects, doubts about efficacy	PubMed/Taylor and Francis
Rehmani and Memon	2010	KSA	Cross-sectional study (*n* = 512 HCWs)	65.6% unvaccinated; main barriers: doubts about efficacy (51.1%), fear of adverse effects (32.1) belief of not being at high risk (27.9%), inconvenience of access (21.7%) fear of injections (18.1%) alternate protection (16%)	PubMed (Vaccine journal)
Awadalla et al.	2020	KSA	Cross-sectional survey (*n* = 312 PHCWs)	45.5% coverage; barriers: fear of side effects (40%), perception that vaccination is not important (24.1%), unsatisfactory past experience (17.6%), lack of knowledge (aOR = 4.22 for non-vaccination)	PubMed/Taylor and Francis
Alzayani et al.	2023	Bahrain	Cross-sectional (*n* = 502 adults, primary care)	Only 36.9% intended to vaccinate; barriers included low perceived severity, preference for natural immunity, doubts about effectiveness, safety concerns, misinformation from social media	Emerald/Arab Gulf J Sci Res
Alsuhaibani	2020	KSA	Multicenter cross-sectional (*n* = 523 HCWs)	48.6% vaccinated; non-adherence due to prior mild influenza (20.7%), young/healthy perception (19.2%), doubts about vaccine effectiveness, unawareness of guidelines, limited training	PubMed/Taylor and Francis
Rabaan et al.	2020	KSA	Questionnaire-based	Uptake increased from 20% (2010) to 44% (2015); barriers included belief vaccine unnecessary and safety concerns	PubMed
Shahbic and Said	2010	Qatar	Cross-sectional (*n* = 9,064 HCWs, Hamad Medical Corporation)	Uptake 19.4%; barriers: lack of time (16.5%), concerns about side effects (13.6%), unawareness of availability	PubMed
Garcell and Ramirez	2014	Qatar	Two vaccine campaigns (2011–2013)	Uptake increased to 61.7 and 71.1%; barriers included lack of awareness, younger staff less likely to vaccinate	PubMed/J Infect Public Health
Abu-Gharbieh et al.	2010	UAE, Kuwait, Oman	Cross-sectional (*n* = 993 HCWs)	Vaccination rates: UAE 24.7%, Kuwait 67.2%, Oman 46.4%; barriers included lack of time (31.8%), inaccessibility (25.4%), concerns about effectiveness (24.9%) and safety (17.3%)	PubMed/Int J Med Sci

KSA, Kingdom of Saudi Arabia; UAE, United Arab Emirates.

#### 3.3.1 Individual factors

It is noted that the barriers commonly cited across the GCC countries include fear of vaccine side effects among 40% unvaccinated HCWs in studies from Saudi Arabia and the United Arab Emirates (22, 34). Misconceptions regarding the severity of the illness, the next most cited cause, was noted for 19.2% of HCWs in Saudi Arabia and 39.8% in Dubai (35, 41). Doubting the efficacy of the vaccine was the next highly cited causes as noted in a quarter of respondents in two studies conducted in Dubai and Saudi Arabia (34, 41). A study conducted across Kuwait, United Arab Emirates and Oman in 2010 revealed that the seasonal vaccination rates were lowest in United Arab Emirates, which was significantly lower compared to Kuwait (67.2%) and Oman (46.4%). The most common reasons cited were lack of time, lack of awareness of the importance and availability of the vaccine and doubts about vaccine efficacy (30).

Studies from Saudi Arabia indicate a wide variation in vaccination rates with the lowest being 34.4%; HCWs cited their hesitancy as stemming from concerns regarding vaccine efficacy and safety, lack of awareness of recommendations by the health authorities and fear of injections (36, 41). A survey in 2016–2017, conducted by the Dubai Health Authority, revealed that 53.4% of primary HCWs were vaccinated, while the remaining respondents reported lack of time (28.9%) and the continuing misconception that their risk of infection was low (39.6%), as reasons for avoiding vaccination (35). Other less commonly cited barriers include fear of needles as reported by 25–40% of nurses and therapists, allergies, and the misconception that the vaccine is useless if there is no influenza outbreak (32). Studies conducted in Qatar in 2010 showed that vaccination rates were as low as 20%, with the most common personal factors for not getting vaccinated being lack of awareness, lack of time and fear of side effects being cited as the most common reasons (38, 39).

#### 3.3.2 Institutional and policy determinants

Fewer institutional campaigns to spread awareness and promote vaccine availability were cited as barriers in a Saudi Arabian study (41). Logistic barriers in terms of convenience of access to vaccination sites and time to receive the vaccine were also frequently cited as reasons to remain unvaccinated as noted by 27.9% of nurses in a study conducted in Abu Dhabi, United Arab Emirates (32). A survey of 502 adults attending primary healthcare facilities in Qatar showed that only 36% were vaccinated. Lack of information, assuming a low risk of infection and preferring natural immunization due to fear of side effects after vaccination, were reported as the most common deterrents to vaccination (40). Garcell and Ramirez studied two consecutive seasons and found that the assumption of having ‘strong immunity’ was a fairly common reason to remain unvaccinated, though the awareness campaigns and vaccination drives were found to improve the vaccination rates (39). A survey of 512 HCWs in KSA found that the inconvenience in accessing the site where vaccination was provided was a deterrent (36).

#### 3.3.3 Sociocultural influences

Misinformation propagated on social media is most common reason for mistrust, especially when compounded with lack of knowledge about the influenza vaccine and belief in the benefits of alternative medicine and lifestyle modulations (34).

Thus, barriers to influenza vaccine among HCWs across GCC countries are rooted in a combination of personal beliefs (fear of side effects, doubts about efficacy, perceived lack of risk), institutional shortcomings (inconvenient timing, insufficient institutional promotion, lack of training), and societal influences that fuel misconceptions (misinformation, reliance on alternative medicine, cultural misconceptions). These challenges highlight that despite vaccines being free and widely available, uptake in GCC countries remains below WHO recommendations of 60–100% for the Eastern Mediterranean countries (42).

## 4 Discussion

It is critical to identify the factors that determine influenza vaccine uptake and assess the progress made in its promotion, because HCWs are a priority group as well as immunization advocates for patients. Influenza vaccination coverage among HCWs across the GCC remains moderate overall but highly heterogeneous. The variation in the vaccine uptake rates among the GCC countries may be attributed to their unique setting, population under study, intensity of the vaccination campaigns and documentation procedures must be considered when comparing the rates. Highest rates are reported in Saudi Arabia during mandatory Hajj vaccination programmes (88%) while the moderate levels in Oman (60%) and United Arab Emirates (54.2%) are an improvement from those reported by earlier studies, while the lowest levels were seen in Bahrain (36.9%) (21, 22, 29, 31, 33, 40). Although the pooled GCC vaccination rate (approximately 51%) exceeds the current global estimate (41.7%), it remains well below the target recommendation for healthcare personnel, indicating persistent gaps in both behavioural acceptance and programme implementation (14–16). The average rates in the other Middle Eastern countries vary from being moderately higher as seen in Jordan (62.8%) to those that are significantly lower as in Iraq (43, 44). These findings indicate that vaccination coverage remains moderate overall in the GCC countries when compared to the other Middle Eastern countries, though with considerable inter-country variability. Comparatively, among the Western countries, successful vaccination drives have a very high vaccine coverage as in Australia (77.9%) and Canada (75.3%) (45, 46). Variations are also noted in the European nations with very high rates in Greece (87.5%) and Spain (78–85%) to lower rates in countries like Slovenia (<40%) and Italy (10.4%) (47–51). The rates in N. America are lower and is on an average is about 44–46% (52). Among the Asian countries a variation from higher rates in Singapore (69.5%) to moderate levels in India (36.8%), Hong Kong (35.6%), Brunei (33.5%), and low levels in countries like Pakistan (8.84%) (53–55). African countries like Tunisia (15.3%) and Sierra Leon (6.5%) account for the lowest global rates of flu vaccination (56, 57).

Using the WHO Behavioural and Social Drivers (BeSD) framework, along with the Confidence, Complacency, Convenience (3Cs) model, the variation observed, across GCC, were interpreted to distinguish psychological determinants of vaccination behaviour from health-system factors that influence vaccine delivery and implementation (58). Accordingly, it suggests that the variability noted is not attributable solely to vaccine hesitancy but reflects the interaction between individual beliefs, workplace culture, organisational policies, and health-system capacity.

### 4.1 Confidence

Across Saudi Arabia, the United Arab Emirates, Oman and Qatar, concerns regarding vaccine safety, fear of adverse effects, and doubts about vaccine effectiveness reduced vaccine uptake despite widespread awareness of influenza (8, 23). Confidence, therefore, seems to be the principal behavioural determinants of influenza vaccination. Similar concerns have been reported internationally, suggesting that confidence remains a universal determinant of vaccination behaviour. The most common barriers in the GCC countries are fear of side effects, doubts about vaccine efficacy and inability to find time to get vaccinated, as reported in 46% of HCWs in Saudi Arabia and 33.6% in Oman (8, 23). These barriers are also cited to deter 48% of HCWs in Tunisia, Singapore, and in Pakistan Jordan, Egypt, Iraq, Spain, and the United States (44, 48, 49, 52, 55, 59–62). However, within the GCC, confidence appears to be closely linked to institutional trust. During coordinated governmental campaigns, particularly those implemented during the Hajj season or national vaccination initiatives, confidence in vaccination increased alongside uptake, indicating that trusted institutional leadership can partially mitigate safety concerns. Trust in governmental authority can act as both an enabler (during mandates) and a barrier (when communication is insufficient), highlighting the dual role of institutional trust in the region (14, 18, 34). Transparent communication strategies highlighting social responsibility while addressing misinformation and leveraging religious values and collectivism may support vaccination efforts effectively by improving confidence more effectively than awareness campaigns alone (14, 18, 34).

### 4.2 Complacency

Complacency also contributed substantially to suboptimal vaccination. An important consideration accounting for the GCC vaccination trends is the high proportion of expatriate HCWs, often from the Asian, African and European countries, which introduces heterogeneity in vaccine perceptions, prior immunization practices, and trust in health systems. Non-national HCWs often demonstrate higher uptake due to prior exposure to stricter vaccination norms or employer-driven compliance, whereas variability among national HCWs may reflect differing risk perceptions and cultural beliefs. This workforce stratification creates intra-system disparities that are less pronounced in more homogeneous healthcare systems globally (11, 34, 37). Across multiple GCC studies, many HCWs perceived influenza as insufficiently severe to justify annual vaccination as they are convinced of having a strong immunity (39). Such perceptions were also reported in studies in Jordan, Egypt, Iraq, Spain, and the United States (44, 48, 49, 61, 62). However, studies also reported HCWs acknowledging self-protection and patient safety as the most compelling individual enablers for up to 95% of United Arab Emirates physicians.

This discrepancy suggests that knowledge alone may not translate into the desired behaviour change. Educational interventions should therefore not only provide information to strengthen risk perception but also emphasize occupational exposure, healthcare-associated influenza transmission, and the ethical responsibility of HCWs to protect vulnerable patients. Behavioural strategies reiterating professional identity and patient safety may therefore be more effective than conventional educational approaches.

### 4.3 Convenience

One of the most important findings was that many barriers reported by HCWs reflect implementation barriers rather than genuine vaccine hesitancy. Although influenza vaccines are provided free to HCWs throughout most GCC countries, numerous studies identified lack of protected time during working hours, inconvenient vaccination schedules, competing clinical responsibilities, and difficulty accessing vaccination services as common reasons for non-vaccination (8, 23, 36). Similar findings were also noted in HCWs in 48% of HCWs in Tunisia and Singapore (52, 59, 60). In Asian countries, like Pakistan, logistical issues like storage and cost were cited (55). In United States additionally, lack of insurance and access to healthcare services are barriers that kept the vaccination rates to 44–46% (52). These reports indicate a willingness to vaccinate but frequently constraints by organisation of the campaigns rather than negative vaccine attitudes. The observed variability across GCC countries, compared to more stable trends in some of the Western healthcare systems, also reflects a unique dependence on episodic, policy-driven interventions (e.g., Hajj mandates) rather than sustained institutionalized vaccination frameworks.

### 4.4 Institutional policy

Mandatory vaccination policies and campaigns conducted by the health authorities like the Saudi Arabian authorities during Hajj season and the PHCC in Qatar are powerful institutional and policy enablers (23, 24, 29, 32); a strategy that is also operational in successful models in Australia, Canada, Greece, Spain and Lebanon (45–47, 63). Educational campaigns to increase awareness, influence exerted by peers and trust inspired in institutions are other enablers that also operate globally (34, 35).

Unlike countries such as Canada and Australia where vaccination is embedded into occupational health requirements, GCC systems often rely on campaign-based approaches, resulting in fluctuating uptake rates across years and settings. Structural integration of vaccination into routine occupational health policy, with accountability, remains less mature in the GCC and must evolve to be embedded into quality and patient safety programmes (11, 44, 45). To strengthen the implementation of the vaccination efforts, GCC healthcare systems would benefit from transitioning from campaign-based strategies to sustained, measurable, policy-driven frameworks to include departmental and organisational vaccination coverage targets annually, flexible clinic hours for protected vaccination time, mobile on-site vaccination carts, mandatory vaccination policies tied to employment, electronic reminders and recall systems, tracking, real time registries for HCW vaccination status integrated with employee health records (64–66). Further, to increase institutional accountability installing departmental vaccination dashboards, updating hospital leadership, incorporating vaccination indicators into quality improvement and accreditation standards, and mandatory documentation of refusal of vaccination and its reasons as declination forms may be beneficial (65–67). Such system-level interventions simultaneously reduce logistical barriers and incorporate vaccination as a routine professional practice. An important discrepancy of higher vaccine uptake among physicians and nurses as compared to other HCWs such as pharmacists, technicians and non-clinical staff, also needs to be addressed by targeted campaigning to ensure adequate coverage (24). Tailored educational materials, involving supervisors and professional associations, and special attention to departments with low coverage may improve vaccine confidence and equity across the healthcare workforce (68). Evidence from GCC studies highlight that multi-component interventions that combine mandates, reminders, and education are more effective than isolated campaigns (11, 14, 65, 66, 68). Additionally, aligning vaccination policies across public and private sectors, in compliance to set guidelines, could reduce variability and improve overall system performance and possibly establish individual attitudes and behaviour in favour of vaccination as an expected professional standard (58, 65, 68).

### 4.5 Social norms and organisational culture

Social norms also emerged as important behavioural drivers. Peer influence, recommendations from supervisors, institutional leadership, and workplace culture consistently reinforced vaccination behaviour across GCC studies (34, 35). In healthcare settings where physicians and senior clinical leaders actively promoted vaccination, uptake was generally higher, reflecting the influence of professional role modelling. The multicultural composition of the GCC healthcare workforce further adds complexity, as expatriate HCWs often bring previous experience of mandatory vaccination policies and differing levels of trust in vaccination programmes, creating an intra-system disparity (11, 34, 37). Consequently, vaccination behaviour may be viewed not only as an individual decision but also as a product of organisational culture and collective professional patterns. In addition to traditional awareness campaigns, influence of respected clinical champions, departmental leadership and peers as role models may therefore strengthen vaccine acceptance.

### 4.6 International comparison

Comparison with international evidence further reinforces the importance of organisational implementation. Countries such as Australia, Canada, Greece and Spain consistently report vaccination coverage exceeding 75%, largely through institutionalised occupational-health programmes supported by mandatory policies, electronic tracking systems, reminder mechanisms, and continuous monitoring (45–51). By contrast, the considerable variation observed across GCC countries appears to reflect reliance on campaign-based approaches rather than mature occupational-health systems. This dependence on episodic interventions may explain the substantial fluctuations observed across institutions, professions, and influenza seasons despite widespread vaccine availability (11, 44, 45).

### 4.7 Implications for policy and practice

Collectively, these findings indicate that improving influenza vaccination coverage within the GCC requires interventions that simultaneously address behavioural determinants and implementation barriers. In the GCC context, barriers are amplified by sociocultural dynamics such as strong reliance on informal information on social media, and culturally embedded beliefs regarding natural immunity and alternative medicine. Trust in governmental authority can act as both an enabler (during mandates) and a barrier (when communication is insufficient), highlighting the dual role of institutional trust in the region (14, 18, 34).

Educational initiatives to primarily target confidence and complacency by addressing misconceptions regarding vaccine safety, efficacy, and occupational risk. Communication strategies highlighting social responsibility, ethical values and collectivism may support vaccination efforts effectively. Reiterating the need for vaccine coverage among those with chronic comorbidities, especially diabetes mellitus, immunocompromised states, chronic inflammatory, chronic kidney disease and malabsorption syndromes, is important for both patients and HCWs. Furthermore, surveillance of HCWs and patients with comorbidities to ensure vaccine coverage (69). Equally important are structural interventions designed to improve convenience, including protected vaccination time, workplace vaccination clinics, mobile vaccination teams, electronic reminder systems, and integrated occupational-health information systems. At the organisational level, consistent vaccination policies across public and private healthcare sectors, embedding vaccination within accreditation standards, and implementing routine electronic monitoring of HCW vaccination status could substantially reduce the vaccine uptake variability (58, 65, 68). Drawing lessons from success of other vaccine preventable viral diseases, such as poliomyelitis, that were effectively controlled and eradicated by building the immunization coverage, mandatory vaccination camp to increase the prevalence of the neutralizing antibodies in the population are essential along with regular surveillance studies (70). Equally important is the educational drives to reinforce the importance of stringent universal safety precautions and disinfection and sterilization procedures to prevent hospital acquired influenza infection, in addition, to hospital protocols and procedures to separate the patients hospitalized during outbreaks from other patients especially those with chronic diseases (71, 72). These efforts should be part of wider institutional strategies to prevent healthcare -associated infections among patients and HCWs. Thus, multicomponent interventions combining policy mandates, education, convenient access, reminder systems and hospital protocols may prove to be more effective than isolated educational initiatives.

### 4.8 Research implications

Despite growing evidence, there remain significant research gaps in the GCC. Most studies were cross-sectional and therefore unable to evaluate sustained vaccination behaviour over multiple influenza seasons. Longitudinal studies assessing sustained vaccine uptake beyond campaign periods are limited, and there is insufficient disaggregated data comparing national versus expatriate HCWs. Additionally, few studies evaluate the effectiveness of digital health interventions (e.g., reminder systems, electronic registries) or behavioural interventions and audit-feedback mechanisms tailored to HCWs. Future research should focus on identification of reasons poor coverage, scalable and context-specific interventions, as well as economic evaluations to guide cost-effective policy decisions in GCC healthcare systems with variability in the available resources (11, 23, 41). Future research should therefore prioritise longitudinal implementation studies, effectiveness–implementation designs, and economic evaluations to determine which interventions or combination of interventions provide the greatest and most sustainable improvements in vaccination coverage in diverse GCC healthcare settings.

## 5 Conclusion

The general uptake of influenza vaccines among HCWs in GCC countries, averages slightly above the global mean but remains significantly heterogeneous and below the WHO’s 75% target. The findings from this review provide insight into ongoing efforts to promote flu vaccination among HCWs. Based on the evidences studied mandatory vaccination programs in institutions were associated with higher coverage compared to voluntary seasonal vaccination. The GCC occupies an intermediate position in the global ranking of influenza vaccine uptake, given its unique context of multinational healthcare workforce and mass gatherings. Variability is noted among different HCW cadres; uptake is generally higher among physicians and nurses compared to pharmacists and technicians. Since self-protection, patient safety, and family safety appear to be the strongest motivators, these enablers should continue to be highlighted during awareness campaigns. On the other hand, concerns regarding side effects and vaccine efficacy, lack of time and access to vaccines, aversion to needles and disbelief in vaccination were the key deterrents to vaccine uptake and should be specifically addressed in these campaigns. Implementation of preventive drives should consider using multiple communication channels for vaccination campaigns, extended availability, free on-site vaccination should be considered to increase vaccine uptake. Cadre-specific attention must be paid when targeting HCWs during influenza vaccination campaigns. This review also provides a foundation for future studies to evaluate investments made for HCWs well-being, and to study vaccination trends to guide the efforts required to mitigate vaccine hesitancy, especially considering the ongoing threat of viral mutations.
